# Letter from New York City

**Published:** 1902-05

**Authors:** 

**Affiliations:** New York


### Domestic Correspondence.

### LETTER FROM NEW YORK CITY.

NEW YORK, April 7, 1902.

TO THE EDITOR:

SIR,—"Some Popular Errors about Microbes" was Dr. Justin De Lisle's subject before The New York Institute of Stomatology's regular monthly meeting, held at Dr. Hoag's office on March 4. A paper of this character coming from one who was associated with Professors Roux and Metchnikoff, Paris, in bacteriological research excited unusual interest. After explaining that there were about seven hundred different species of microbes, out of which forty-one were considered pathogenic germs to the animal kingdom and thirty-one dangerous to man, he pointed out some of the erroneous notions regarding microbes, also the protection which unabraded skin afforded against them. He dwelt at some length regarding the employment of serums for the prevention of danger from these microbes, and also the possibility of the employment of serums in the future to combat diseases of microbic origin. He spoke of the advisability of using the antitoxin tetanus serum in instances where fear of infection from tetanus bacilli indicated its use at the outset of suspected infection, for, as the doctor said, the tetanus germ being developed from the spore, or seed, as it were, required a certain number of days to develop after the wound was healed or closed to the light and air, hence the extreme importance of early inoculation of the antitoxin serum in indicated instances.

Experiments were cited where guinea-pigs which from their birth were reared in a sterilized atmosphere, with sterilized food, only survived thirteen days, here pointing out the important part played by bacteria in preservation of life itself. He also pointed out the danger of over-antiseptic treatment, wherein the surrounding tissues under such treatment were robbed of their natural recuperative powers, thus retarding the healing process. The paper was ably discussed by Dr. Hopkins, of Boston, and others, and the vital question as to the proper disinfection of dental instruments was discussed. A one to five per cent. of carbonate of soda in boiling water to prevent rusting was recommended by Dr. Dawbarn as a safe, economic, and reliable method. Other important matters were discussed, after which the meeting adjourned and a collation was served.

On March 11 Dr. Goldan, of New York, read a paper before the First District Dental Society, on "Nitrogen Monoxide in Combination with Oxygen for Anæsthesia." His method of administration was carefully explained. The advantage claimed for the combination over the plain nitrogen monoxide was that of doing away with cyanosis, and consequently more natural and normal breathing during the operation; the percentage of oxygen used was from eight to fourteen per cent. of nitrogen monoxide. The short tube was preferred in the administration, for Dr. Goldan thought that irritation of the respiratory tract was in a measure due to the respective gases passing from the cylinders through the long tube to the mouth. Among those who took part in the discussion was Dr. Hasbrouck, who, after commending the excellent features in the paper, still believed in the use of nitrogen monoxide with the atmospheric air. Dr. Hatch, in favorably considering the subject of discussion, stated that its practice had been pursued both here and abroad by others, and also that chloroform as an anæsthetic was considered by the medical profession as being preferable to ether in its administration in regard to children. Others followed in the discussion.

A resolution passed by the National Dental Association in 1900, in relation to examination of school children's teeth, was acted upon, this society co-operating with the State Dental Society in the matter. The meeting then adjourned.

Dr. F. T. Van Woert, of Brooklyn, read a practical paper, illustrated with stereopticon views, before the New York Odontological Society, on March 18, at their regular monthly meeting, in which was clearly demonstrated the necessary steps taken for the production of the X-ray picture; also setting forth the great value of the X-ray in diagnosing cases in certain instances. A subject of the practical value of this, and requiring so much illustration in the description of it, is only truly appreciated by personal attendance at a meeting, as those present upon the above occasion would testify. Suffice it to say, the X-ray has come to stay in the dental profession, and as Dr. Morton, in speaking later in the evening, remarked, the possibilities of the X-ray as a therapeutic agent are very great, and no doubt marked developments along this line in this direction may be looked for in the future. The progress that has already been made with some wonderful results in the therapeutic action of the X-ray was discussed. The intense burns arising under certain conditions, with the slow process of healing, due undoubtedly to the effect of the X-ray upon the arteries or blood-vessels in affected parts, was also brought forth, Dr. Dwight M. Clapp, of Boston, and others joining in the discussion, after which the meeting adjourned.

LOCHINVAR.

